# The rs361525 polymorphism does not increase production of tumor necrosis factor alpha by monocytes from alpha-1 antitrypsin deficient subjects with chronic obstructive pulmonary disease - a pilot study

**DOI:** 10.1186/s12952-015-0039-3

**Published:** 2015-12-01

**Authors:** Jennie M. Gane, Robert A. Stockley, Elizabeth Sapey

**Affiliations:** Institute of Inflammation and Ageing, University of Birmingham, Edgbaston, Birmingham, B15 2TT UK; Lung Investigation Unit, University Hospital Birmingham NHS Foundation Trust, Edgbaston, Birmingham, UK; Centre for Translational Inflammation Research, New Queen Elizabeth Hospital, University of Birmingham Laboratories, Institute of Inflammation and Ageing, Edgbaston, Birmingham, B15 2TT UK

**Keywords:** TNF-α, COPD, Monocytes, Polymorphism, Alpha-1 Antitrypsin Deficiency

## Abstract

**Background:**

Polymorphisms in the TNF-A gene have been associated with chronic obstructive pulmonary disease (COPD) in some case-control studies. Previous work has shown that COPD/chronic bronchitis subjects with alpha-1 antitrypsin deficiency with the rs361525 TNF-α single nucleotide polymorphism have 100 times more TNF-in spontaneous sputum than disease matched controls. Our objective was to determine if the presence of this polymorphism increased TNF-α production by blood monocytes from COPD subjects.

**Findings:**

Monocytes from 18 COPD/alpha-1 antitrypsin deficient subjects, with and without the rs361525 polymorphism, were cultured in the presence or absence of lipopolysaccharide. Cell-free supernatants were analyzed by ELISA and real-time PCR performed using cDNA from extracted RNA. Baseline expression of TNF-α messenger RNA was no different between the groups. No difference in messenger RNA or secreted protein was observed over time in un-stimulated cells. TNF-α messenger RNA expression and protein was not higher in lipopolysaccharide-stimulated monocytes from subjects with the polymorphism compared to cells from patients with the wild-type allele.

**Conclusions:**

This small pilot study did not provide an explanation for the findings of earlier observations of the association of the rs361525 polymorphism with TNF-α in airways secretions. Possible reasons for the lack of concordance include the study of blood rather than tissue cells, the use of a single stimulant rather than biological secretions and the need for far greater subject numbers to overcome intra-subject variation in monocyte TNF-α production.

## Findings

### Introduction

Chronic obstructive pulmonary disease (COPD) is a debilitating disease most commonly caused by smoking. However, only 25 % of smokers develop COPD, suggesting other contributing factors, such as genetic susceptibility [1]. The only well described example of genotype influencing the development of COPD is Alpha-1 Antitrypsin Deficiency (AATD), characterised by deletions/substitutions within the serpinA1 gene. The resultant reduction of neutrophil elastase-buffering alpha-1 antitrypsin leads to the development of airflow obstruction and emphysema [2].

There is a pressing need to identify new therapeutic targets in COPD, to modify disease and improve outcomes. COPD is heterogeneous and it is possible that there are many potential targets, each affecting a subset of patients. One such target may be tumor necrosis factor-alpha (TNF-α) which has been implicated in the pathogenesis of COPD in animal studies [3, 4] and observational human studies [5]. Whilst TNF-α is not ubiquitously raised in COPD [6] and anti-TNF-α treatments are not efficacious in a generalised COPD population [7], single nucleotide polymorphisms (SNPs) in the TNF-A gene have been identified that might enhance susceptibility to COPD by increasing inflammatory output. Wood *et al* characterised a cohort of 424 unrelated patients with COPD and AATD and determined that the rs361525 polymorphism, a G to A substitution at position -238 in the promoter region of the gene, was observed with greater frequency in subjects with a chronic bronchitis phenotype [8]. Analysis of spontaneous sputum samples from 10 subjects revealed 100 times greater soluble TNF-α concentration compared to matched controls, suggesting the polymorphism may affect gene transcription. In addition, in lung secretions there was a higher concentration of downstream pro-inflammatory mediators, greater decline in lung function and lower BMI, indicating a more aggressive disease phenotype. No difference in serum concentration of TNF-α was observed and was low in both groups which may reflect rapid binding to tissue receptors preventing its detection [9].

Alpha-1 antitrypsin is involved in the modulation of TNF-α biosynthesis and AATD patients are known to have increased activation of the TNF-α pathway [10]. Therefore patients with both AATD and a pro-inflammatory TNF-α SNP might be more exposed to TNF-α inflammation, enhancing the cellular and clinical effects of the polymorphism.

We hypothesised that monocytes (a principal TNF-α producing cell) from subjects with AATD and COPD would produce more TNF-α if they carried the rs361525 polymorphism and that this would have an enhancing downstream effect on other monocyte functions relevant to COPD, such as phagocytosis and cytokine production. As no previous experiments had been conducted in monocytes with the rs361525 polymorphism from this patient group, it was not possible to power the current studies and hence the work presented here should be considered as a pilot study.

## Methods

This study was conducted following ethical approval from the National Research Ethics Service Committee West Midlands, UK (reference number LREC-3359/3359-A). Patients with AATD and COPD were recruited from the UK AATD registry, held in Birmingham, UK, following the provision of written informed consent. All patients were clinically stable and exacerbation free for at least six weeks prior to recruitment. Patients were selected following careful matching for clinical criteria (described in Table 1). Subjects with the polymorphism are referred to as AG and those with the wildtype allele, GG. Monocytes were extracted using the Dynabeads® Untouched™ Human Monocytes kit (Life Technologies, Paisley, UK). Cells were re-suspended in culture medium (sterile Roswell Park Memorial Institute medium 1640 (Sigma Chemicals Ltd, Poole, UK) supplemented with 10 % fetal calf serum, 10%L-glutamine and 10 % penicillinV and streptomycin and cultured at 37 °C and in 5 % CO_2_. Monocytes were plated at a concentration of 0.25 (for mRNA experiments) or 0.45 million per ml (in duplicate for ELISA experiments) of culture medium. Salmonella Enteritidis derived lipopolysaccharide (LPS) (100 ng/ml; Sigma Chemicals Limited, Poole, UK) was chosen as the TNF-α stimulant after conducting concentration-response and time-course experiments in healthy control monocytes to confirm it elicited the greater response, compared to a number of other stimuli, with a peak time point for TNF-α protein (6 h post LPS). An enzyme-linked immunosorbant assay (ELISA) was used to measure TNF-α in the cell-free supernatant according to manufacturer instructions (R&D Systems, Abingdon, UK). The plates were read using a Synergy HT microplate reader (Biotech, GMI, Ramsey, USA). All samples and standards were run in duplicate. mRNA was extracted from each cell pellet using the Isolate RNA Minikit (Bioline, London, UK). Median 260/280 ratio of RNA samples was 2.1 (IQR 1.8-2.6). RNA samples were reverse transcribed using a High Capacity RNA-to-cDNA Kit (Life Technologies, Paisley, UK) in a Takara Thermal Cycler PCR machine (Takara Bioeurope, Saint-Germain-en-Laye, France). Real-time quantitative polymerase chain reaction (PCR) was carried out to quantify the expression of each gene of interest. Complementary deoxyribonucleic acid (cDNA) was mixed with Light Cycler 480 Probe PCR Master master mix (Roche Applied Science, Burgess Hill, UK), PCR-grade water and the relevant fluorescein isothiocyanate-labelled TaqMan gene expression assay (Life Technologies, Paisley, UK), in each well of the plate. The reaction was run on a Roche Lightcycler 480 (Roche Applied Science, Burgess Hill, UK), for 45 amplification cycles. The Assay-on-Demand numbers for the TaqMan assays were: glyceraldehyde 3-phosphate dehydrogenase (GAPDH): Hs99999905_m1; TNF-α: Hs00174128_m1. Where an individual normalising gene can be shown to be stably expressed in the model under study it is deemed acceptable to use only that one [11]. Stable reference genes have been classified as those in which the average fold change from the mean expression was less than 2 and the maximum variability in fold change less than 5 [12]. GAPDH was found to be stably expressed in a random selection of 64 samples (from different subjects and under a variety of experimental conditions) with a mean fold change from the mean CT value of 1.7 and a maximum fold change of 4.6. Singleplex reactions with equal starting quantities of cDNA were conducted. Samples were run in duplicate and the average of two cycle threshold values taken. The 2^-ΔCT^ formula was used to calculate the relative expression of mRNA [13]. Data is presented as median and IQR and differences between groups tested with a Mann Whitney U test. Data was analysed using the SPSS statistical program (version 20.0 Chicago, USA).

**Table 1:** Characteristics of study subjects

Characteristic		rs361525 + ve (AG/AA)	rs361525 -ve (GG)	P value (2- tailed)
N		9	9	
		(8 AG/1 AA)		
Presence of COPD		9/9	9/9	
Age in years		51 (50–65)	60 (49–61)	0.8
Male		7 (77.8)	7 (77.8)	
AATD level (micromolar)		3.8 (0.4)	4.0 (0.5)	0.8
BMI		21.2 (21.0–23.8)	23.2 (22.0–26.0)	0.3
Smoking hx	Current	1	0	
	Ex/never	7/1	8/1	
Pack years		22 (10–28)	22 (9–30)	0.9
FEV1 (L)		1.4 (0.1)	1.4 (0.2)	0.8
FEV1 % predicted		37.7 (33.4–43.4)	33.6 (32.6–54.6)	0.9
FEV1/FVC ratio (%)		32.0 (28.0–34.0)	32.2 (23.0–33.0)	0.5
KCO % predicted		53.9 (4.6)	52.0 (4.8)	0.8
Emphysema on HRCT		8/9	9/9	
Chronic bronchitis phenotype		4/9	5/9	
Bronchiectasis on HRCT		4/9	5/9	
Inhaled steroids		8/9	9/9	
Median exacerbations per year		1.0 (1.0–2.0)	0.5 (0–1.0)	0.3

The table shows key characteristics of subjects with (AG/AA) and without (GG) the rs361525 TNF-α polymorphism. Subjects were matched as closely as possible. Data is given as mean (SE) where normally distributed and median (IQR) where not normally distributed. An independent t-test was used to detect any difference between groups for the former data and a Mann Whitney U test for the latter

## Results

Table 1 shows the characteristics of study subjects. There were 9 subjects in each group. Data for mRNA work was available for only 8 subjects in each group (due to a technical issue in mRNA extraction for one subject). Patients were matched closely, as shown in Table 1. There were no statistically significant differences between the groups.

**Fig. 1:**
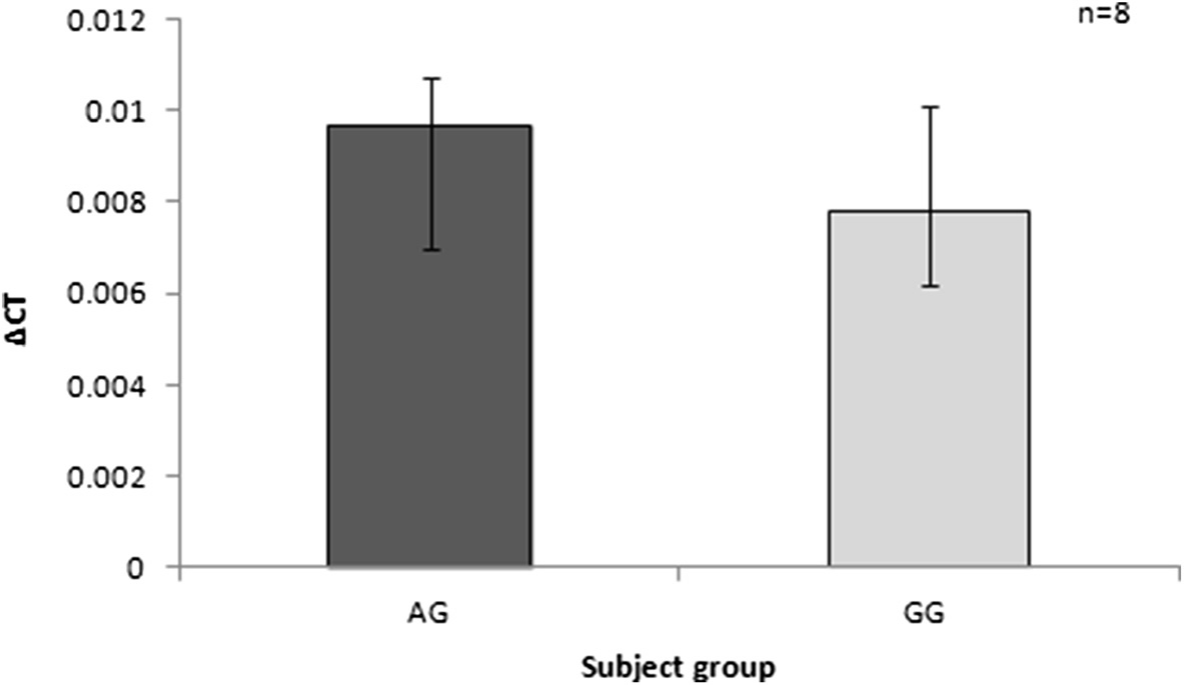
Baseline expression of TNF-α mRNA in freshly isolated monocytes from patients with and without the rs361525 TNF-α polymorphism. Columns show median (IQR) ∆CT values for TNF-α mRNA expression (normalised to GAPDH). Differences between subject groups were assessed with a Mann Whitney U test. There was no significant increase in TNF-α mRNA expression in the AG monocytes

**Fig. 2:**
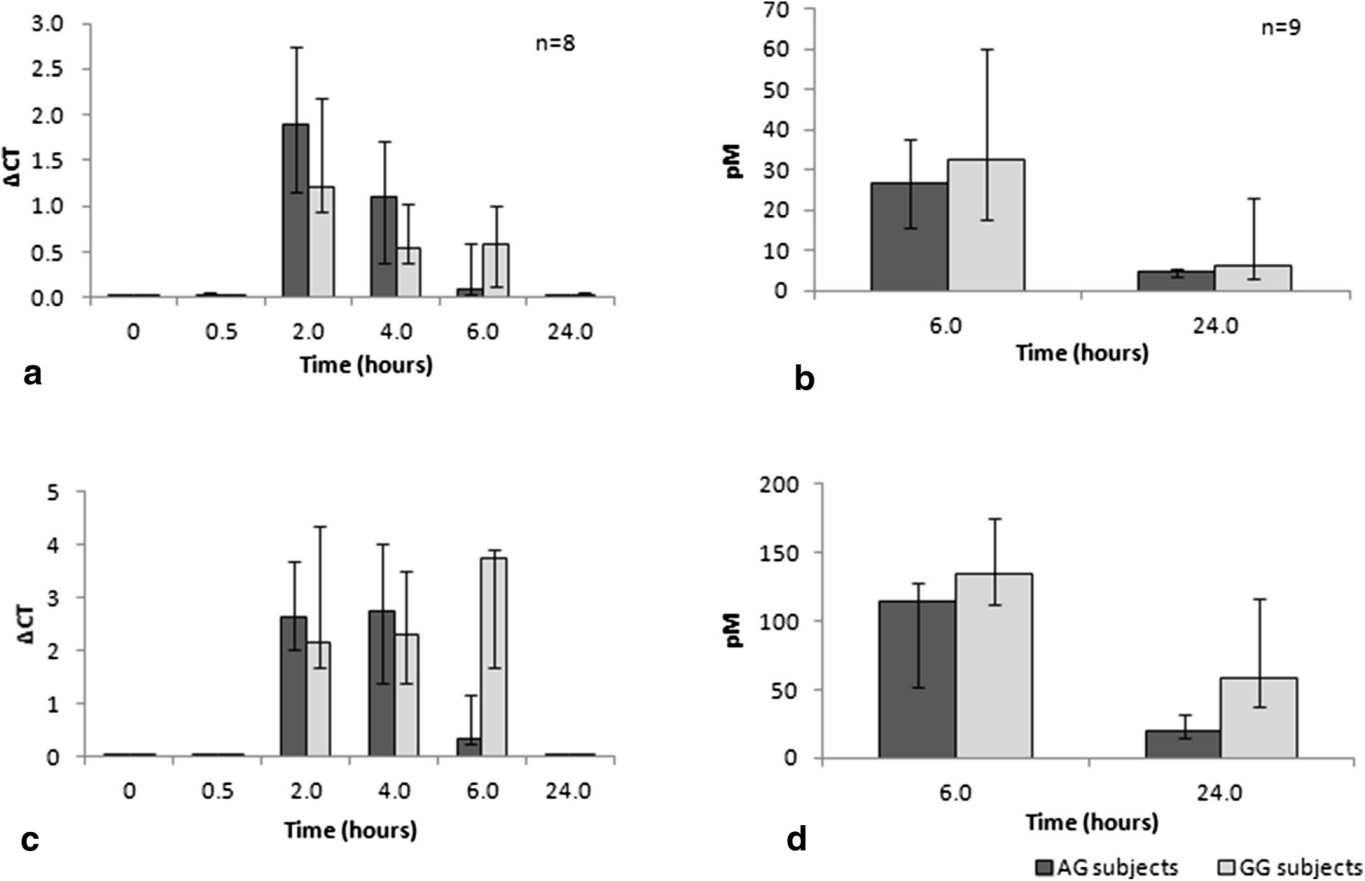
TNF-α production over time by monocytes from patients with and without the rs361525 TNF-α polymorphism. **a** ∆CT values for TNF-α mRNA expression (normalised to GAPDH) in un-stimulated monocytes cultured over 24 hours. **b** Concentration of sTNF-α in the supernatant of un-stimulated monocytes cultured for 6 and 24 hours. **c** ∆CT values for TNF-α mRNA expression in LPS-stimulated monocytes cultured over 24 hours. **d** Concentration of sTNF-α in the supernatant of LPS-stimulated monocytes cultured for 6 and 24 hours. Results are displayed as median (with IQR). There was no significant increase in TNF-α mRNA expression or protein concentration in the AG monocyte group

**Fig. 3:**
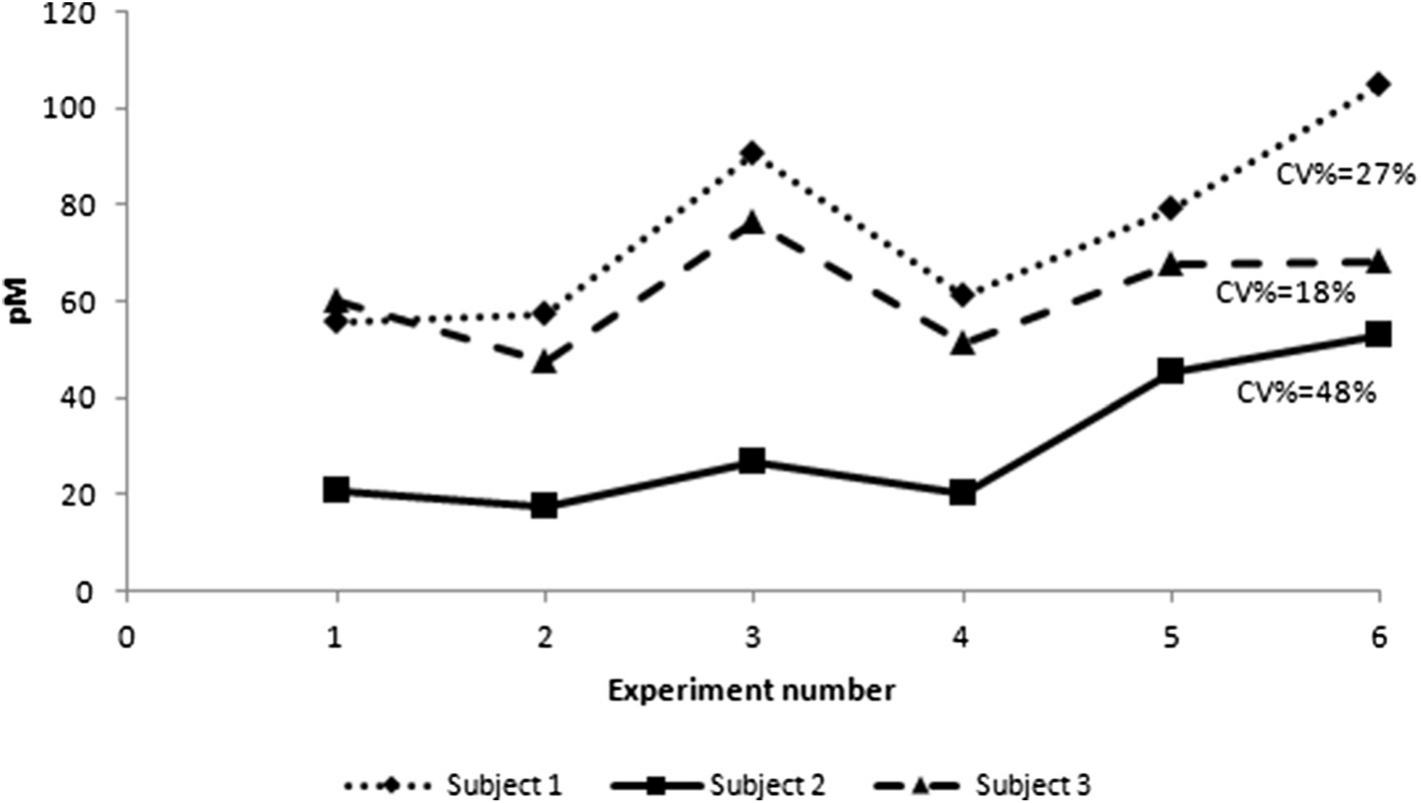
TNF-α secretion by LPS-stimulated monocytes over three weeks. Monocytes from 3 healthy subjects (2 female) were isolated twice weekly for 3 weeks and stimulated in duplicate with 100 ng/ml of LPS for 3 hours. TNF-α concentration in the cell-free supernatant was measured using ELISA. Coefficient of variation (CV%) values for each subject are shown

Expression of TNF-α mRNA in freshly isolated un-stimulated monocytes was low and there was no difference between AG and GG subjects (Fig. 1). No difference in mRNA expression or secreted protein was observed between the two groups in monocytes cultured without any stimulus (Fig. 2a and b). LPS-stimulated monocytes isolated from AG patients did not show more mRNA expression or protein concentration compared to the GG (wild type) (Fig. 2c and d). We hypothesized that measuring TNF-α on one occasion may be insufficient to determine a true difference between groups, should intra-subject variation in TNF-α secretion over time be high. Monocytes from 3 healthy subjects were therefore isolated twice weekly (3-4 days apart) for 3 weeks, stimulated with 100 ng/ml of LPS, and TNF-α concentration in the cell-free supernatant measured at 3 h. Figure 3 shows the values over 3 weeks for each subject. Coefficient of variation percentage values for subjects one to three were 26.9, 48.4 and 17.7 %. These data were used to calculate that a sample size of 40 patients per AG/GG group would be required to demonstrate a true 20 % difference in sTNF-α concentration in the supernatants six hours post LPS stimulation with approximately 80 % power.

## Discussion

The pilot studies presented here did not support the findings in the sputum of COPD/AATD patients with the rs361525 polymorphism, which was surprising given the magnitude of difference observed in the airways [9]. It is clear from subsequent experiments studying the intra-subject variation in TNF-α that large numbers of subjects in each group would be required to have adequate power to detect even a modest difference in production should this exist in monocytes in relation to this stimulus and hence further studies were not conducted.

There are other possible explanations for the lack of expected effect. Although LPS has been shown to induce transcription of TNF-α mRNA in immortalised cells of the monocyte lineage with the AG genotype using a reporter gene assay [14], the same study demonstrated that multiple hits are required to maximally potentiate the effects of the polymorphism. Specifically, binding of thyroid hormone receptor to the A allele in addition to LPS-induced nuclear factor kappa beta was necessary. It is possible that the complex cytokine milieu in the lungs of COPD patients is such that multiple mechanisms are in place to enhance TNF-α mRNA transcription in subjects with the A allele. By the same rationale it may be that the effects of the polymorphism are specific to the airways of COPD/AATD patients with chronic bronchitis, due to specific local stimulating factors, again potentially reducing the power if this is the critical disease phenotype. In the current study only 50 % of subjects had chronic bronchitis in addition to emphysema, and these low numbers prevented further analysis of any clinical subgroup. Finally, monocytes may not be the relevant cell to study. The increased TNF-α concentration in the sputum might reflect output specifically from macrophages, T-cells or bronchial epithelial cells. Indeed, the original study found no difference in systemic levels of TNFα between groups perhaps suggesting this is a compartment specific finding [9].

In summary our findings did not support our primary hypothesis but are consistent with the literature describing the effects of this particular polymorphism. Studies report positive, negative and no effects, in a range of cells types, employing a wide variety of techniques to measure output and in different disease states [9, 14–25]. This study reflects the inherent difficulty in studying the effects of SNPs at a cellular level and we suggest further investigation of the rs361525 SNP should focus on airway derived cells and local transcription factors.

## Abbreviations

AATD: Alpha-1 Antitrypsin Deficiency
BMI: Body mass index
cDNA: Complementary deoxyribonucleic acid
COPD: Chronic obstructive pulmonary disease
CV%: Coefficient of variation percentage
ELISA: Enzyme-linked immunosorbant assay
FEV1: Forced expiratory volume in 1 second
FVC: Forced vital capacity
GAPD: Glyceraldehyde 3-phosphate dehydrogenase
HRCT: High resolution computed tomography
KCO: Transfer coefficient
LPS: Lipopolysaccharide
mRNA: Messenger ribonucleic acid
PCR: Polymerase chain reaction
SNP: Single nucleotide polymorphism
TNF-α: Tumour necrosis factor-alpha
